# Tapered Fibers Combined With a Multi-Electrode Array for Optogenetics in Mouse Medial Prefrontal Cortex

**DOI:** 10.3389/fnins.2018.00771

**Published:** 2018-10-26

**Authors:** Leonardo Sileo, Sebastian H. Bitzenhofer, Barbara Spagnolo, Jastyn A. Pöpplau, Tobias Holzhammer, Marco Pisanello, Filippo Pisano, Elisa Bellistri, Emanuela Maglie, Massimo De Vittorio, Patrick Ruther, Ileana L. Hanganu-Opatz, Ferruccio Pisanello

**Affiliations:** ^1^Istituto Italiano di Tecnologia, Center for Biomolecular Nanotechnologies, Arnesano, Italy; ^2^Developmental Neurophysiology, Institute of Neuroanatomy, University Medical Center Hamburg-Eppendorf, Hamburg, Germany; ^3^Department of Microsystems Engineering (IMTEK), University of Freiburg, Freiburg im Breisgau, Germany; ^4^ATLAS Neuroengineering bvba, Leuven, Belgium; ^5^Dipartimento di Ingegneria dell’Innovazione, UniversitaÌ del Salento, Lecce, Italy; ^6^Cluster of Excellence BrainLinks-BrainTools, University of Freiburg, Freiburg im Breisgau, Germany

**Keywords:** optogenetics, optrode, optical fibers, medial prefrontal cortex, tapered fibers

## Abstract

Optogenetics offers many advantages in terms of cell-type specificity, allowing to investigate functional connectivity between different brain areas at high spatial and neural population selectivity. In order to obtain simultaneous optical control and electrical readout of neural activity, devices called “optrodes” are employed. They are typically composed of a linear array of microelectrodes integrated on a slender probe shafts combined with flat-cleaved optical fibers (FF) placed above the recording sites. However, due to tissue absorption and scattering, light delivered by the FF unevenly illuminates the region of interest. This issue is of particular relevance when cellular populations are disposed along the dorso-ventral axis, such as in medial prefrontal cortex (mPFC) where cortical layers are aligned vertically. The study presented here aims at using tapered optical fibers (TFs) in combination with a 16-electrode neural probe to better access neural populations distributed along the dorso-ventral axis in the mPFC of newborn mice, restricting light delivery over a specific portion of the cortical layer of interest. Half of the TF surface is coated with a reflecting metal blocking the light to enable light delivery from one side of the probe’s shaft only, with the probe base being designed to host the fiber without interfering with the wire-bonds that connect the recording sites to a printed circuit board. Monte-Carlo simulations have been implemented to define the relative TF-probe position and to identify the light intensity distribution above the recording sites. *In vivo* recordings indicate that simultaneous optical stimulation and electrical readout of neural activity in the mPFC benefit from the use of the engineered TF-based optrode in terms of a more uniform light distribution along the dorso-ventral axis and the possibility of restricting light delivery to a subset of electrical recording sites of interest.

## Introduction

With the increasing use of optogenetics to investigate functional connectivity in the mouse brain, the development of implantable devices for the simultaneous optical control and electrical monitoring of neural activity has been a major research focus in recent years (Grosenick et al., 2015; Cho et al., 2016; Pisanello et al., 2016). In their earlier implementation, these opto-electrodes (optrodes) were composed of a single light source and a single recording electrode (Gradinaru et al., 2007). More than 10 years of development have allowed obtaining different configurations, in which multiple optical stimulation channels can be accompanied with multi electrode arrays (MEA), providing multi-point optical control and electrical readout of neural activity. This can be obtained with several technologies, including μLEDs realized on one substrate comprising multiple recording sites (Wu et al., 2015) or on separate substrates (Ayub et al., 2016, 2017), flexible electronics (Kim et al., 2013; Goßler et al., 2014) or solid state waveguides (Segev et al., 2016; Schwaerzle et al., 2017; Lanzio et al., 2018) potentially providing multiple diffraction gratings for the outcoupling of light (Lanzio et al., 2018).

**FIGURE 1:**
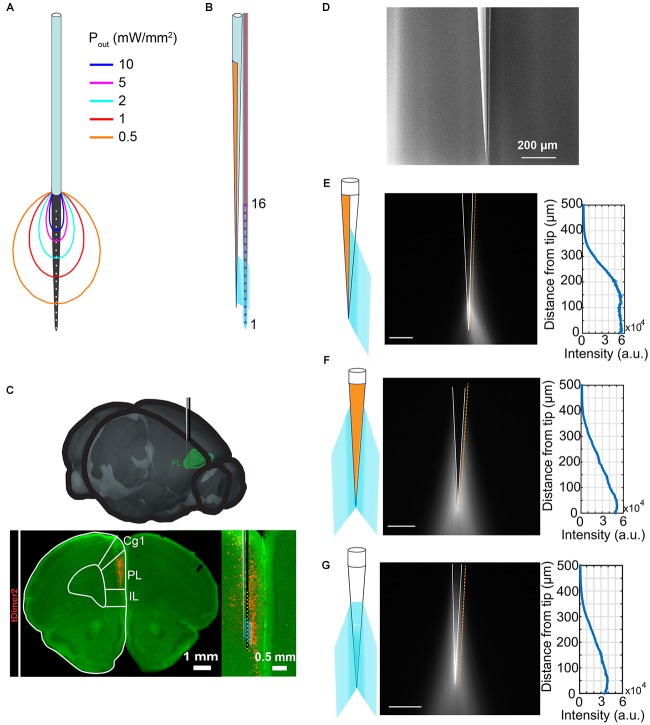
Tailoring light delivery to a subregion of mPFC. **(A)** Representation of a commercially available optrode (A1 × 16-3mm-100-703-OA16LP, light fiber terminates 200 μm from the top recording site) and power density distribution iso-lines obtained with Monte-Carlo simulations for a total power of 1 mW delivered to the tissue. **(B)** Schematic representation of the optrode designed in this work, consisting of a half-metallized TF beside a multielectrode array. **(C)** Schematic overview of recording setup of the prelimbic subdivision of the mPFC. Digital photomontage reconstructing the position of the designed opto-electrode in the PL of a P9 mouse after IUE with ChR2(ET/TC) and tDimer2 (red) at embryonic age (E)15.5. Inset, optical light fiber (gray) and electrode shank (black) including the position of the recording sites (white) over the prelimbic depth are displayed at higher magnification. **(D)** Typical scanning electron microscope image of the realized TF. **(E–G)** Emission properties of the realized TF (side view) in a cartoon (left) and with the TF submerged in fluorescein:PBS droplet (center). Half of the taper is coated with ∼5 nm of Cr and ∼200 nm of Au. The graph (right) shows the emission profile acquired along the orange line in the center panel (scale bars represent 100 μm).

Although these technologies have the potential to help neuroscientists to better match stimulation and recording patterns with the anatomy of the brain region of interest, devices commonly used in neuroscience labs are still based on flat-cleaved optical fibers (FF) placed above linear arrays of electrodes for extracellular recording (Neuronexus, 2015; Atlas Neurotechnologies, 2017; Cambrige Neurotechnologies, 2017). However, this widely used approach encounters important limitations when the cellular population of interest is distributed along the dorso-ventral direction. Indeed, light emitted above the recording sites undergoes tissue attenuation and scattering. This results in a highly inhomogeneous distribution of power density, that can span several orders of magnitudes along the recording sites if they are positioned along more than 1 mm (Yizhar et al., 2011; Stujenske et al., 2015; Schmid et al., 2016). This fact is illustrated in Figure 1A with a Monte-Carlo simulation indicating the power density in brain tissue generated by an optical fiber with a numerical aperture NA = 0.22 and a core size of 100 μm, emitting light above a linear array of 16 electrodes. As indicated by the iso-power density lines, one obtains a decrease in optical power by about two orders of magnitude from the top most recording site to the bottom one. The above-mentioned technologies based in microsystems engineering can help in challenging this issue by placing multiple emitters very close to the individual recording sites. However, these discrete sets of light delivery points face different pitfalls, such as a potential tissue heating induced by Joule’s effects for μLEDs, limited outcoupling efficiencies of diffraction gratings and the high commercialization costs to make these probes available to neuroscience labs. In this scenario, tapered optical fibers (TFs) (Pisanello et al., 2014, 2017) represent on the other hand a valid alternative to these approaches, allowing to tailor the light delivery pattern to the anatomy of the functional region of interest (Pisanello et al., 2018; Pisano et al., 2018). This is possible by exploiting two main features of these devices: (i) the narrowing waveguide allows to exploit mode division demultiplexing to deliver light gradually along a specific segment of the taper (Pisanello et al., 2018); indeed, as the taper narrows, the number of guided modes supported by the waveguide decreases, with modes not allowed to propagate toward the tip being outcoupled around the taper, and (ii) the possibility of using metal coatings to mask emission and to direct light to specific sites and directions (Pisano et al., 2018).

In this work we describe the engineering of an optrode based on a TF placed besides a 16-electrodes silicon-based neural probe following the Michigan style. The tapered fiber is designed to deliver light to the tissue above a subset of recording sites by exploiting a gold coating deposited on one half of the taper. Monte-Carlo simulations are implemented to define the relative TF-shank placement to obtain a fairly uniform power density in the tissue above the selected electrodes. The geometrical assembly is specifically thought to deliver light on (and to record signal from) pyramidal neurons in layers 2/3 of the prelimbic (PL) region of the medial prefrontal cortex (mPFC), in which Channelrhodopsin (ChR2) was selectively expressed by means of *in utero* electroporation (IUE). Extracellular electrophysiology data in mice at postnatal day (P) 8–10 confirm that the device can be used to monitor both local field potentials and single unit action potentials, with the TF design allowing for a more uniform illumination above the chosen subset of recording sites.

## Results

### Optrode Design and Fabrication

The optrode presented in this work has been designed with the goal of engineering light delivery in the mPFC of rodents. Area- and layer-specific stimulation in prefrontal networks is critical to understand the origin and significance of neuronal activity patterns. Layer-specific optogenetic stimulation in the mPFC can be achieved by layer-specific expression of ChR2 by IUE (Figure 1C) (Bitzenhofer et al., 2017a,b). However, the restriction of transfection to specific subdivisions of the mPFC is difficult to achieve with *in utero* electroporation, but critical to study prefrontal function due to different or even opposing functions of dorsal and ventral subdivisions of the mPFC (Hardung et al., 2017). The alternative approach to restrict stimulation is to limit the area of illumination in transfected tissue. However, most commercially available optrodes are often based on linear array of electrodes for extracellular recording combined with a FF placed above the probe shank, as illustrated in Figure 1A. This configuration results in a highly inhomogeneous illumination of neurons directly facing the recording sites, as quantified by the displayed iso-power density lines, obtained with the Monte-Carlo method published by Stujenske et al. (2015) to assess the power density distribution along the electrode carrying probe shank.

To overcome this limitation and to obtain a more uniform light intensity across specific recording sites, we engineered an optrode design consisting of a silicon-based probe in combination with a TF emitting light from one side of the silicon shank (Figure 1B). The optical design of the TF is optimized with the aim of providing a relatively uniform light intensity only in a region above selected recording sites along the probe shank. Further, the light emission geometry matches the dorso-ventral extension of the neural population of interest. Among the available TFs configurations (Pisano et al., 2018), we chose an optical fiber with core/cladding diameters of 105 μm/125 μm and a numerical aperture of NA = 0.22. The TF was realized by the heat-and-pull technique, resulting in a taper angle Ψ of ∼4°, which has already been shown to provide a tissue illumination over an extent of ∼400 μm (Pisanello et al., 2018). To deliver light only toward the shank, half of the taper is coated with a 200-nm-thick, thermally evaporated gold layer. A typical output of the fabrication process is shown in the scanning electron microscope image in Figure 1D, while the light delivery behavior of a TF with these geometrical characteristics is illustrated in Figures 1E–G for three different angular views, with the TF submerged in a fluorescent liquid. By virtue of the metal reflectivity, light delivery is confined to about 180° around the waveguide.

**FIGURE 2:**
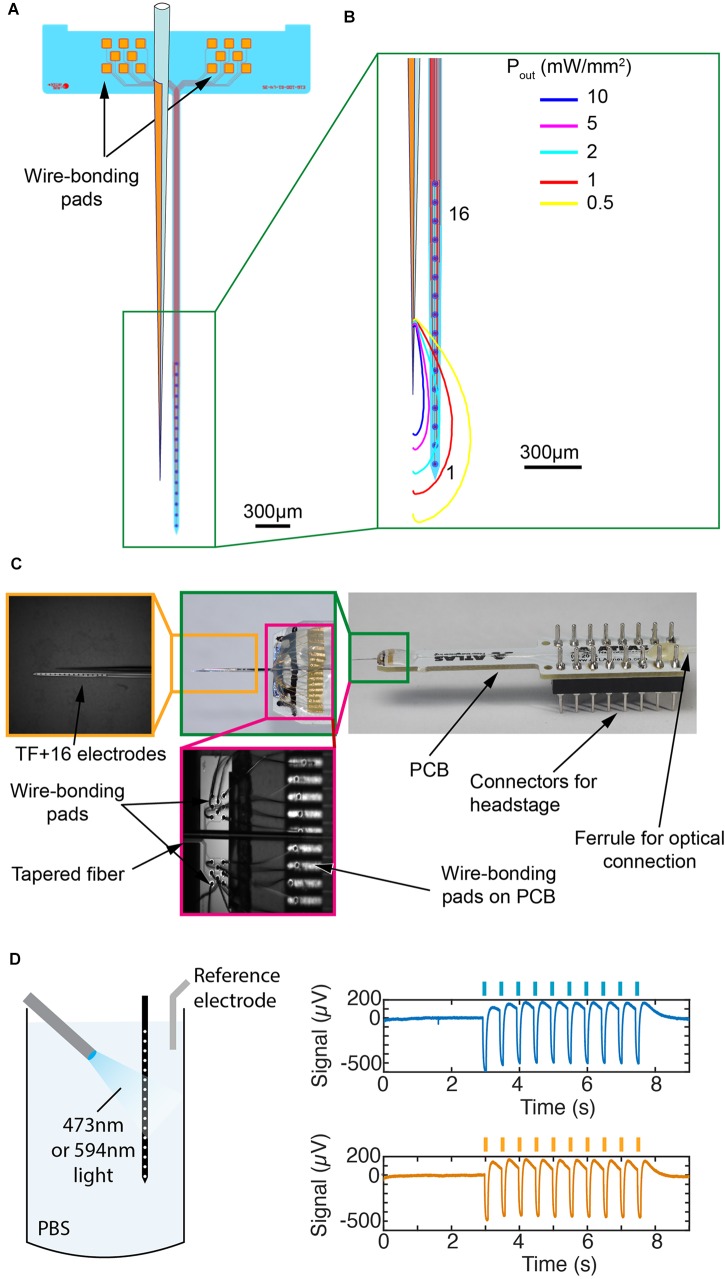
Engineering of the TF-based optrode. **(A)** Layout of the silicon-based probe array with 16 IrO*_*x*_* recording sites (diameter 35 μm) arranged at a pitch of 100 μm along a 4-mm-long probe shank and two groups of bonding pads arranged on the probe base (2.5 mm × 0.58 mm). Fiber position is also reported. **(B)** Monte-Carlo simulations of power density distribution iso-lines generated by the realized TF and overlapped with a sketch of the microelectrodes array. **(C)** Final optrode assembly on a PCB (right side) with enlarged view of the wire bonds on the probe base and PCB (center) and the tip region indicating the electrodes and TF (left). **(D)** Test of photoelectric effect induced by blue or yellow light in PBS. An optical fiber was placed 900 μm in front of the shank and delivers 200-ms-long pulses of 10 mW at wavelengths of 473 or 594 nm. The two graphs on the right show photoelectric effects generated by both wavelengths (the non-filtered signal is shown). Peak-to-peak amplitudes are 687 μV ± 13 μV and 624 μV ± 8 μV (mean ± std, *n* = 10) for the 473 and 594 nm lasers, respectively.

The neural probe applied here (see probe layout in Figure 2A) comprises a slender, tapered probe shaft with a maximum width of 75 μm at the probe base. The shaft carries 16 recording sites with a diameter of 35 μm arranged at a center-to-center distance of 100 μm. The probe shaft is connected to a rectangular probe base (2.5 mm × 0.58 mm) on which two groups of eight contact pads (80 μm × 80 μm) are arranged, interfacing the individual recording sites. The probe metallization is made of a layer stack of titanium/gold/titanium with the electrode metallization being realized by reactive sputter deposition and lift-off of iridium oxide. Probe shaft and base have a thickness of 50 μm and are realized using standard micro-electromechanical systems (MEMS) technologies combined with the etching before grinding (EBG) approach (Herwik et al., 2011).

To estimate light delivery properties in scattering tissue and to define the relative TF-shank placement, a modified Monte-Carlo algorithm was implemented to take into account light emission along a tapered waveguide. This approach allows to estimate light distribution around the taper and to engineer the relative position between the TF and the probe shank. Figure 2B shows iso-power density lines for a TF which is placed at ∼150 μm beside the shank emitting a total optical power of 1 mW. Tissue above the electrodes #1 through #6 receive a relatively uniform power density of about 2 mW/mm^2^. Recording sites #8 to #16 receive an optical power density < 0.5 mW/mm^2^, with a steep decrease across electrode #7. This represents a key difference between the TF-based optrode design and the commercially available system shown in Figure 1A, in which tissue above electrodes from #7 to #16 receive a power density above 2 mW/mm^2^, but in a highly inhomogeneous way.

In order to position the TF within the above-mentioned distance relative to the probe shank, the electrical contact pads on the probe base interfacing the recording sites on the shank are laid out as depicted in Figure 2A, i.e., the two groups of eight pads are positioned off-center on the probe base with respect to the probe shaft position. This allows for wire bonding the pads to a printed circuit board (PCB) while the optical fiber is positioned in-between the two groups of contact pads. The resulting optrode is shown in Figure 2C, assembled by using two computer-controlled micromanipulators to obtain a precise relative positioning and axial alignment between TF, probe shank and PCB. Once in place, the TF is adhesively fixed to the probe base using UV curable epoxy glue, which at the same time mechanically protects the wire bonds between the pads on the probe base pads and PCB. The optical microscopy image in Figure 2C illustrates the highly parallel alignment between TF and probe shank expected to minimize tissue damage during optrode insertion into the brain.

### Optical Control and Electrical Readout of Neuronal Activity in the mPFC of Neonatal Mice

**FIGURE 3:**
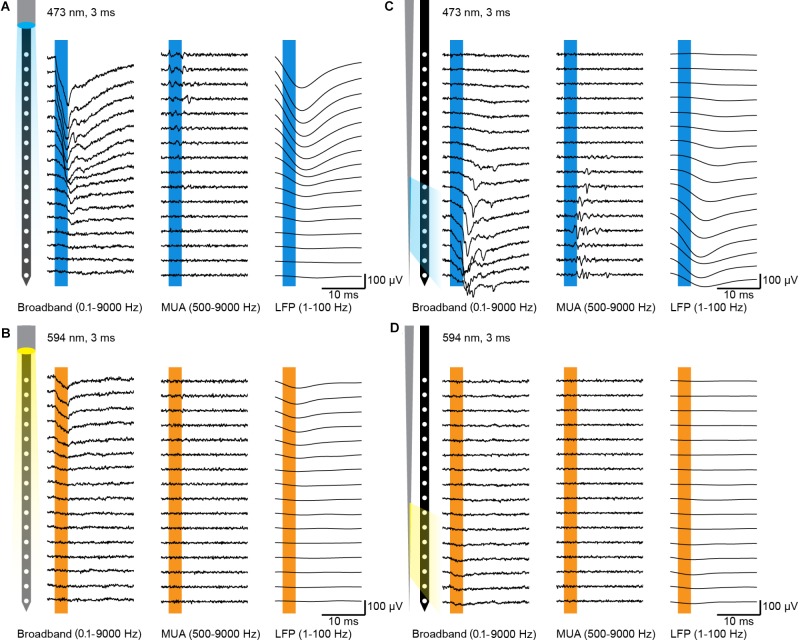
Typical electrophysiology recordings with FF- and TF-based optrodes. **(A)** Characteristic extracellular multi-site recording of light-induced activity (3 ms pulse, 473 nm) with illumination from the top in the mPFC of a neonatal mouse expressing ChR2(ET/TC) in layer 2/3 pyramidal neurons transfected by IUE. **(B)** Same as A for stimulation with yellow light (3 ms pulse, 594 nm) to trigger pure light artifact. **(C,D)** Same as A,B for illumination from the side with a TF.

**FIGURE 4:**
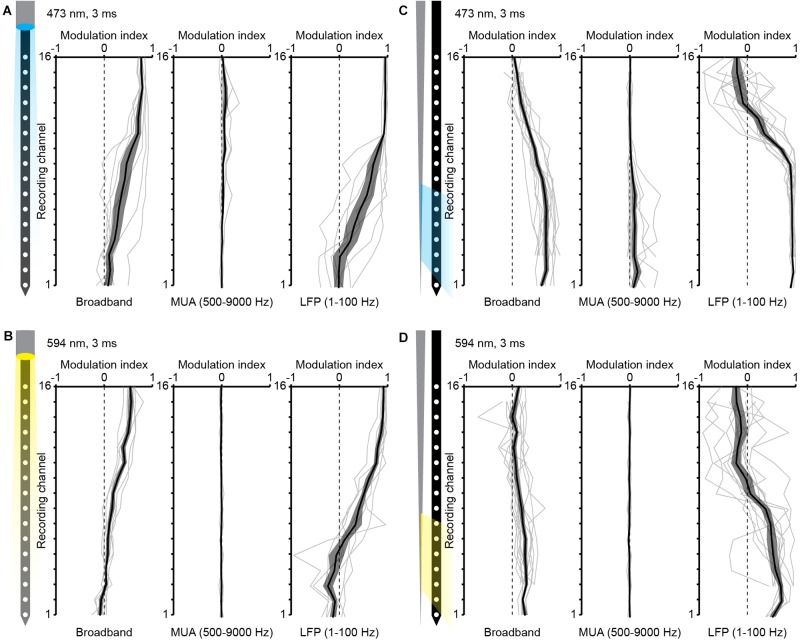
*In vivo* experiments over multiple animals. **(A)** Quantification of the modulation index over recording sites for illumination with blue light pulses (3 ms, 473 nm) with a commercial optrode with illumination from the top (*n* = 7 mice). **(B)** Same as A for illumination from the side with a TF (*n* = 12 mice). **(C)** Same as A with yellow light (3 ms, 596 nm) to estimate photoelectric response. **(D)** Same as C to estimate photoelectric response of TF-based optrodes.

Light fibers on commercially available optrodes typically end above the top recording sites, i.e., the electrode positioned closest to the probe base. With this configuration light can be easily restricted to local patches of cortex at the surface of the brain, such as somatosensory or motor cortices. The mPFC is located at the midline of the forebrain with vertically oriented layers. With the available optrodes it is hard to restrict the inhomogeneous illumination of cortical patches to prefrontal subdivisions, especially for small sized neonatal mouse brains. In order to evaluate whether the TF-based optrode engineered in this work overcomes these limitations, we compared it to a commercially available FF-based optrode (NeuroNexus, A1 × 16-3 mm-100-703-OA16LP, light fiber terminates 200 μm from the top recording site, Figure 1A) by using *in vivo* optogenetics in neonatal mice. For extracellular recordings the optrodes were inserted (2.4 mm) into the mPFC to reach ChR2-expressing pyramidal neurons in layers 2/3 of the prelimbic subdivision in mice at the age of (P) 8–10 (Figure 1C). Laser power for light delivery was adjusted to trigger neuronal spiking in response to > 20% of 3 ms-long light pulses at 16 Hz. Typical single channel local field potentials (LFPs, right column) and multi-unit activity (MUA, middle column), defined as activity in the frequency band from 500–9000 Hz, are displayed in Figures 3A–D for FF and TF optrodes, respectively. TF and FF induced neural activity was compared in response to blue light pulses (473 nm, total output power ∼1 mW, Figures 3A,C). Blue light pulses evoked comparable LFP and MUA for light delivery with TF and FF at the recording sites close to the fiber output, with the TF optrode eliciting neural activity on about 7 recordings sites starting from the tip, as expected from the Monte-Carlo simulations shown in Figure 2B. To characterize light artifacts of the probes and to distinguish them from induced activity in the same position where light-triggered activity is measured, yellow light pulses (594 nm, Figures 3B,D) that do not activate ChR2 were used. *Ex vivo* recordings of photoelectric effects with pulsed light of 473 and 594 nm induced similar light artifacts (Figure 2D). To evaluate the evoked responses over multiple animals, a modulation index was defined as (*x*_stim_ −*x*_pre_)/(*x*_stim_ + *x*_pre_), where *x*_stim_ is the median amplitude of the broadband signal, LFP or MUA in response to the light pulse and *x*_pre_ is the median signal amplitude before the pulse. Results of this analysis are reported in Figure 4. For both LFP and MUA channels, top illumination with FF evoked strong activity at the topmost recording sites with waning modulation toward the shaft’s tip, whereas illumination with TF triggered activity mainly in the illuminated recording sites, as shown in Figures 4A,C, respectively. Compared to FF, TF-based optrodes illuminate a subset of recording sites more homogenously resulting in more evenly distributed activity. Light artifacts recorded with yellow light stimulation were present in the frequency band of LFP, but not in MUA for TF and FF, as shown by the single channel data in Figures 3B,D and multiple animal averages in Figures 4B,D. Thus, TFs provide sufficient light output to trigger comparable activity in mPFC of neonatal mice as FF, with TF allowing for a more homogenous illumination of local cortical patches.

## Discussion and Conclusion

In this work we propose an optrode design thought to better distribute light on cellular populations distributed in the dorso-ventral direction and to restrict illumination to subregions of the mPFC. It is based on a half-coated TF placed beside a 16-electrode silicon-based neural probe, whose relative position allows for delivering light to brain tissue addressed by a subset of the 16 recording sites. The system was tested on neonatal mice expressing ChR2 in layers 2/3 pyramidal neurons in the mPFC, distributed dorso-ventrally over ∼2 mm, as shown in Figure 1C. To reduce the invasiveness of the optrode, also because of the mice’s young age at the time of the experiment, we chose to work with the smallest-available TF (NA = 0.22, core/cladding diameters 105 μm/125 μm, taper angle Ψ ≈4°, sub-micrometer tip). FF commonly used to reach this region, have diameters of 125 μm causing increased tissue damage. They generate light-stimulated activity over ∼7 recording sites (Figure 3A) with a highly inhomogeneous power density, ranging from 20 mW/mm^2^ to 2 mW/mm^2^, exponentially decreasing as the distance from facet increases (Figure 1A). Moreover, the geometrical configuration of FF-based optrodes has an intrinsic limitation related to fiber positioning: electrodes close to the shaft’s tip interfacing with the less-damaged tissue can hardly be reached with a high-enough optical power density, unless light power delivered to the fiber is increased by two orders of magnitude (Schmid et al., 2016). Instead, placing a side-emitting TF beside the neural probe allows to direct light on the tissue above the selected recording sites. Although the data reported here are shown for illumination of recording sites close to the shank’s tip, the TF/probe design enables using any subset of adjacent recording sites by changing the relative position of the TF along the probe shaft. To define the set of illuminated recording sites, a Monte-Carlo simulation approach was implemented, allowing to identify the geometrical distribution of photons and the above-threshold region prior to the experiment. This, coupled with the possibility to engineer light delivery geometries by tailoring the taper angle (Pisanello et al., 2018) or using micro and nano fabrication approaches to structure the fiber taper (Pisanello et al., 2014, 2015; Pisano et al., 2018; Rizzo et al., 2018), let us envision that the TF-based optrode configuration can be extended to several experimental designs.

The activation of cells adjacent to specific subsets of recordings sites can be obtained also by other technologies, including optrodes based on μLEDs and solid-state waveguides. However, these approaches are based on different technological platforms that require complex fabrication processes, highly increasing the costs and time to bring them to market. Our design is instead based on a well-established combination between optical fibers and silicon-based neural probes following the Michigan style, and it requires only a few process steps to be modified: (i) layout of the wire-bonding pads on the probe base and (ii) precise alignment and assembly of the TF relative to the probe shaft.

TF-based optrodes provide a better control of neuronal activity, due to homogenous illumination of several recording sites induced by the highly different distribution of light typically seen for FF-based optrodes. They also improve spatial restriction of illumination to local patches of vertically oriented cortical areas. The ability to homogenously illuminate locally restricted patches in vertically oriented cortices, such as the mPFC, allows to investigate functions and interactions of prefrontal subdivisions, such as the prelimbic and infralimbic PFC throughout development. Overall, the optrode configuration proposed in this work allows for a better access to dorso-ventrally distributed neuronal populations, improving light delivery uniformity and to choose the position and the number of the recording sites to be illuminated.

## Materials and Methods

### Realization of the Multi-Electrodes Probe

The silicon-based electrode array, as illustrated in Figure 2A with the respective mask layout, is realized using the etching before grinding (EBG) technology detailed elsewhere (Herwik et al., 2011). It applies 4-inch silicon (Si) wafers which are covered on their front side with a stress-compensated 1-μm-thick layer stack of silicon-oxide (SiO*_*x*_*) and silicon nitride (Si*_*x*_*N*_*y*_*) realized by plasma enhanced chemical vapor deposition (PEVCD). Next, the probe metallization which interfaces the recording sites with the contact pads via 1.5-μm-wide metal tracks is deposited and patterned using sputter deposition of titanium (Ti, 30 nm), gold (Au, 250 nm) and Ti (30 nm) and lift-off applying an image reversal resist, respectively. The metallization is covered by another, stress-compensated PECVD stack of Si*_*x*_*O/Si*_*x*_*N*_*x*_* (total thickness 1.5 μm). This layer stack is patterned using reactive ion etching (RIE) in combination with a positive photoresist serving as the masking layer to electrically access the contact pads (80 × 80 μm^2^) on the probe base and the recording sites through respective vias (diameter 5 μm). This etch step removes as well the upper Ti layer exposing the Au of the probe metallization. Next, the electrode metallization (diameter 35 μm) is deposited by sputter deposition and reactive sputter deposition of iridium (Ir, 100 nm) and iridium oxide (IrO*_*x*_*, 200 nm), respectively. The Ir/IrO*_*x*_* layer stack is patterned using lift-off as well. Subsequently, we pattern the dielectric PECVD layer stacks (total thickness 2.5 μm) using RIE followed by deep reactive ion etching (DRIE) of the bulk silicon substrate to generate trenches (width 40 μm) defining to probe shape. These trenches are etched to a depth *t*_etch_ exceeding the intended probe shaft thickness *t*_shaft_ by 20 μm. Finally, the silicon wafer is ground from the rear using a commercial grinding process by DISCO Hi-TEC Europe GmbH (Kirchheim, Germany) to a thickness of 50 μm by which the probes are automatically released. Once peeled from an adhesive tape used during wafer grinding, the probes are ready for assembly. For this, probes are adhesively fixed onto a PCB and wire bonded using gold wires with a diameter of 25.4 μm.

### Tapered Fibers Fabrication and Assembly With the Neural Probe

Tapered fibers were obtained from OptogeniX^1^ with a taper angle of ψ ≈ 4° and a numerical aperture of NA = 0.22 (core/cladding diameters 105/125 μm) (Pisanello et al., 2018). A 5-nm-thick Cr layer and a 200-nm-thick gold layer were deposited along the taper using evaporation which blocks ca. 180° of the TF from light emission. In order to ensure a correct deposition of the layers also close to the tip of the TF, the fiber was slightly tilted toward the crucibles during evaporation. After metal deposition, the obtained fibers were connectorized with a ceramic ferrule (diameter 1.25 mm) resulting in an overall fiber length that matches with the neural probe-PCB assembly (Figure 2C). Optical properties of the waveguide were tested in a PBS:fluorescein bath and emission profiles were determined by recording the fluorescence counts on a line parallel to the taper edge (Figures 1E–G).

The as prepared TF is fixed on a micromanipulator (Scientifica) and placed in parallel to the probe shank at a distance of ∼100 μm aligning the TF tip with the fifth electrode. The non-tapered fiber section is positioned in the space between the two groups of probe base bonding pads and fixed with UV curable epoxy glue.

### Monte-Carlo Simulations

Monte-Carlo simulations were implemented in Matlab to estimate the power density distribution generated by flat-flat cleaved fibers (Figure 1A) or TFs (Figure 2B). In the case of FFs, the method proposed by Stujenske et al. (2015) was used to model an optical fiber with NA = 0.22 and core/cladding diameters of 105/125 μm, emitting a total power of 1 mW. In the case of metal-coated TFs, the code described in Stujenske et al. (2015) was modified in order to account for light emission from the conical surface of the taper. This was implemented by modifying the initial conditions of emitted photons in terms of emission position and output angles. Photon emission probability along the taper was estimated from the direct measurement of the light emission profile (see measurement in Figure 1E), while output angles were obtained by ray tracing simulations (Pisanello et al., 2018). Around the taper axis, photons emission probability was considered uniform in the angular range 0° to 180° and zero from 180° to 360°, to simulate the presence of the metal layer. Scattering was simulated in a domain of size 3 mm × 3 mm, discretized with a mesh of 5 μm × 5 μm. The matrix resulting from the simulation represents a spatial distribution of photons, weighted in intensity according to the energy left in a steady state (Stujenske et al., 2015). To draw the iso-power density lines in Figures 1A and 2B, a 2-dimensional filter and a threshold were applied according to the examined power density volume. Output power was set to 1 mW and the Henyey–Greenstain scattering model was used at α = 473 nm, with absorption coefficient *a* = 0.37 mm^−1^, scattering coefficient *s* = 11 mm^−1^ and anisotropy parameter *g* = 0.89.

### *In utero* Electroporation

All experiments were performed in compliance with the German laws and the guidelines of the European Community for the use of animals in research and were approved by the local ethical committee (Behörde für Gesundheit und Verbraucherschutz/Lebensmittelsicherheit und Veterinärwesen) (132/12, N18/015). Timed-pregnant C57Bl/6J mice were housed individually in breeding cages at a 12 h light/12 h dark cycle and fed *ad libitum*. Vaginal plug detection was defined embryonic day (E) 0.5, while birth was assigned as postnatal day (P) 0. Additional wet food supplemented with 2–4 drops Metacam (0.5 mg/ml, Boehringer-Ingelheim, Germany) was given from 1 day before until 2 days after surgery. At E15.5 pregnant mice were injected subcutaneously with buprenorphine (0.05 mg/kg body weight) 30 min before surgery. Surgery was performed under isoflurane anesthesia (induction: 5%, maintenance: 3.5%) on a heating blanket, eyes were covered with eye ointment, and toe pinch reflex and breathing were monitored throughout the surgery. Uterine horns were exposed and moistened with warm sterile PBS. 0.75–1.25 μl solution containing 1.25 μg/μl DNA pAAV-CAG-ChR2(E123T/T159C)-2A-tDimer2 and 0.1% fast green dye were injected in the right lateral ventricle of each embryo using pulled borosilicate glass capillaries. Each embryo was placed between the electroporation tweezer-type paddles (5 mm diameter, Protech, TX, United States) oriented at a 20° leftward angle from the midline and a 10° angle downward from anterior to posterior to transfect neural precursor cells of layer 2/3 medial prefrontal pyramidal cells. Five electrode pulses (35 V, 50 ms, 950 ms interval) were applied with an electroporator (CU21EX, BEX, Japan). Uterine horns were placed back into the abdominal cavity after electroporation and abdominal muscles and skin were sutured.

### *In vivo* Tests

Multi-site extracellular recordings were performed in the mPFC of P8-10 mice. Mice were injected i.p. with urethane (1 mg/g body weight; Sigma-Aldrich, MO, United States) prior to surgery. Under isoflurane anesthesia (induction: 5%, maintenance: 2.5%) the head was fixed into a stereotaxic apparatus using two plastic bars mounted on the nasal and occipital bones with dental cement. The bone above the PFC (0.5 mm anterior to bregma, 0.1 mm right to the midline for layer 2/3) was carefully removed by drilling a hole of < 0.5 mm in diameter. After a 10–20 min recovery period on a heating blanket, linear multi-site optrodes with a flat-cleaved light fiber attached ending 200 μm above the first recording site (NeuroNexus, MI, United States), or linear multi-site neural probes with a tapered light fiber attached were inserted 2.4 mm deep into the mPFC perpendicular to the skull surface. A silver wire in the cerebellum served as ground and reference electrode. Extracellular signals were band-pass filtered (0.1–9000 Hz) and digitized (32 kHz) with a multi-channel extracellular amplifier (Digital Lynx SX, Neuralynx, Bozeman, MO, United States) and the Cheetah acquisition software (Neuralynx, Bozeman, MO, United States). Pulsed light stimulations were performed with an arduino uno (Arduino, Italy) controlled laser (473 nm/594 nm, Omicron, Austria). Recording signals were band pass filtered to isolate local field potential (LFP, 1–100 Hz) and multi-unit activity (MUA, 500–9000 Hz) using a third-order Butterworth filter forward and backward to preserve phase information. To reduce signal contamination by photoelectric effects, 10-ms-long time windows starting 1 ms after the end of the light pulse were analyzed.

## Data Availability Statement

The datasets for this study are available upon request to the authors.

## Author Contributions

All authors listed have made a substantial, direct and intellectual contribution to the work, and approved it for publication.

## Conflict of Interest Statement

TH had an academic [Department of Microsystems Engineering (IMTEK), Albert-Ludwigs-Universität Freiburg, Germany] and company (ATLAS Neuroengineering bvba, Leuven, Belgium) affiliation. The remaining authors declare that the research was conducted in the absence of any commercial or financial relationships that could be construed as a potential conflict of interest.

## Footnotes

1 www.optogenix.com
